# Identifying Volar Locking Plates on Plain Radiographs: Can Artificial Intelligence Models ‘Beat’ Clinicians?

**DOI:** 10.7759/cureus.98715

**Published:** 2025-12-08

**Authors:** Allen Albert, Alex Nicholls, Duncan Avis, Medhat Zekry, Matay Arsan, Priyanshu Saha, Adam Stoneham

**Affiliations:** 1 Trauma and Orthopaedics, Homerton University Hospital, London, GBR; 2 Trauma and Orthopaedics, Hampshire Hospitals NHS Foundation Trust, Hampshire, GBR

**Keywords:** artificial intelligence, image recognition, implant identification, volar locking plate, x-ray analysis

## Abstract

Background

Hand surgeons are frequently required to identify volar locking plates on plain radiographs. This is important, for example, when planning what equipment is required for revision, implant removal or periprosthetic fractures, but it can be challenging, especially if surgery took place in another hospital or even country. Artificial intelligence (AI) clearly has potential in medical image recognition, but its role in orthopaedic implant identification currently remains uncertain. This study compared the performance of an openly available AI model, ChatGPT 5, with that of experienced hand consultants.

Methods

Fifty-two radiographs of distal radius plates from 10 major implant manufacturers were obtained from open-access sources. An AI programme (ChatGPT 5) and five hand consultants independently identified the manufacturer for each radiograph. Accuracy was calculated for each rater. Pairwise comparisons between AI and each consultant were assessed using McNemar’s test, and a standard logistic regression with clustered standard errors was fitted to compare AI with consultants as a group.

Results

ChatGPT5 correctly identified just 3 of 52 radiographs (5.8% accuracy). Consultant accuracies ranged between 13.5 and 46.2% (mean 30.8% ± 11.1). McNemar’s test showed that Consultants 1, 3, 4 and 5 significantly outperformed the AI (p < 0.01), while Consultant 2 did not (p = 0.289). In a standard logistic regression with clustered standard errors, the human cohort had 7.26 times higher odds of correct identification compared with the AI (OR 7.26, 95% CI 2.27-23.18, p < 0.001).

Conclusion

Identifying volar locking distal radius plates from plain radiographs remains difficult, even for experienced surgeons and even the ‘best’ consultant identified under 50% correctly. That notwithstanding, humans were on average seven times better than ChatGPT 5 which only identified just over 5% correctly. While current non-specialised AI tools are not suitable for implant identification currently, dedicated AI models trained on curated orthopaedic datasets may hold promise for future clinical use.

## Introduction

Distal radius fractures rank amongst the commonest adult orthopaedic injuries; the incidence has been steadily rising over the last 20 years, and this trend is expected to continue [1]. Treatment can be nonoperative or operative depending on many factors including the degree of displacement and the patients’ age and comorbidities [2], while significant variation exists in clinical practice [3]. Although good results are achievable with percutaneous wire fixation [4,5], there has been a steady rise in the use of internal fixation in North America [6,7] and Europe [8,9] and volar locking plates have risen from around 20% of surgeries in 1997 to over 90% in 2018 [10].

Hardware removal following open reduction and internal fixation is controversial with wide variation in practice between different surgeons in different countries [11]. Although there is little support for routine removal in asymptomatic patients, justifiable indications include pain, stiffness, or irritation and the procedure is successful and straightforward in many cases [12,13]. Other indications for removal might include revision or in the instance of periprosthetic fractures [14].

To remove the plate and screws, it is necessary to know what hardware was used during the index procedure and, in particular, what implant-specific equipment (e.g. screwdriver) will be required. This can be challenging, especially if the surgery took place in another institution or country, in emergency settings, or if the operative notes are not available. Very little evidence exists regarding surgeons’ ability to determine the make and model of distal radius plates based on plain radiographs alone. Artificial Intelligence (AI) may already have a role in detecting distal radius fractures [15,16]. We postulate that it will also have a growing role in helping surgeons in this situation, thereby potentially reducing operative time and complications.

## Materials and methods

Fifty-two AP radiographs of in-situ distal radius plates were obtained from open-access online sources to obviate issues surrounding copyright or patient confidentiality. The images included 10 major international manufacturers, with at least five radiographs per brand to ensure balanced representation. Each radiograph was independently verified against manufacturer catalogues and online implant databases to confirm brand identity. The included manufacturers and their parent companies are listed in Table 1.

**Table 1:** Distal radius plate manufacturers, parent companies, and countries of origin

Manufacturer	Parent company/Corporate group, Headquarters
Acumed	Colson Medical / Marmon Holdings (Berkshire Hathaway), USA
Arthrex	Arthrex, Inc. (independent), USA
DePuy Synthes	Johnson & Johnson MedTech, USA
KLS Martin	KLS Martin SE & Co. KG, Germany
Skeletal Dynamics	Skeletal Dynamics LLC (independent), USA
Newclip Xpert	Newclip Technics, France
Smith & Nephew	Smith & Nephew plc, UK
Stryker	Stryker Corporation, USA
Medartis	Medartis AG, Switzerland
Zimmer Biomet	Zimmer Biomet Holdings, Inc., USA

An openly available artificial intelligence model, ChatGPT 5 (paid version), OpenAI, USA was asked to correctly identify the manufacturer of each distal radius plate. The same set of radiographs (in a random order) was also distributed to five orthopaedic hand consultants from two different hospitals in England, who were independently asked to identify the manufacturer of the plates.

All responses, both from ChatGPT and from the consultants, were recorded and scored using a binary coding system: 1 for a correct identification and 0 for an incorrect identification. Statistical analysis was performed using R (version 4.5.1), with a significance threshold set at p < 0.05. Accuracy was calculated for each rater. For pairwise comparisons between each consultant and AI, McNemar's test was performed to report the matched odds ratio. To compare AI performance with all consultants combined, a standard logistic regression with clustered standard errors was performed to report odds ratios (ORs) and 95% confidence intervals (CIs).

## Results

Five consultant hand surgeons took part in this study with a total of 34.5 years of consultant experience (range 1 - 12, mean 6.9, Standard Deviation 3.9).

Across the 52 radiographs, the AI programme correctly identified three implants (5.8% accuracy) while the five hand consultants (Consultants 1, 2, 3, 4 and 5) achieved accuracies of 38.5% (20/52), 13.5% (7/52), 46.2% (24/52), 28.8% (15/52), and 26.9% (14/52), respectively. The mean consultant accuracy was 30.8% (SD 11.1; range 13.5 - 46.2%) (Table 2).

**Table 2:** Accuracy of AI model and five independent hand consultants in identifying volar locking distal radius plate manufacturer (n = 52 radiographs) Accuracy was calculated as the proportion of correctly identified plates. Descriptive statistics for human raters (Consultants) include the mean ± standard deviation (SD) and the observed range (minimum–maximum).

Rater	Correct / (n)	Accuracy (%)
AI programme	3/52	5.80%
Consultant 1	20/52	38.50%
Consultant 2	7/52	13.50%
Consultant 3	24/52	46.20%
Consultant 4	15/52	28.80%
Consultant 5	14/52	26.90%
Consultants (mean ± SD)	–	30.8% ± 11.1
Range (min–max)	–	13.5 – 46.2%

Pairwise comparisons using McNemar’s χ² test demonstrated that four of the consultants significantly outperformed the AI (Table 3).

**Table 3:** Pairwise comparison of AI vs individual consultants using McNemar’s χ² test Cross-tabulation of correct and incorrect responses for the AI programme versus each consultant across 52 radiographs. “Both Correct” and “Both Wrong” indicate cases where the AI and the consultant produced identical outcomes. “AI Correct / Consultant Wrong” and “AI Wrong / Consultant Correct” show discordant cases and these were used to create contingency tables for McNemar’s analysis. The matched odds ratio (OR) quantifies the relative odds of correct identification by the consultant compared with the AI. Statistical significance was evaluated with McNemar’s χ² test with continuity correction (df=1) and it was used to compare correct identification rates between the AI and each consultant. Both the χ² statistic and p-value are shown. Statistical significance was defined as p < 0.05 (corresponding to χ² ≥ 3.84).

Comparison	Both Correct	AI Correct/Consultant Wrong	AI Wrong/Consultant Correct	Both Wrong	Matched OR	χ²(1)	p-value
AI vs Consultant 1	3	0	17	32	∞	15.06	<0.001
AI vs Consultant 2	1	2	6	43	3	1.13	0.289
AI vs Consultant 3	2	1	22	27	22	17.39	<0.001
AI vs Consultant 4	0	3	15	34	5	6.72	<0.01
AI vs Consultant 5	2	1	12	37	12	7.69	<0.01

When all human consultants were considered together, a standard logistic regression with clustered standard errors accounting for repeated measures within radiographs showed that humans had 7.26 times higher odds of correct identification compared with the AI programme (OR 7.26, 95% CI 2.27 - 23.18, Wald z 2.97, p < 0.001) (Table 4).

**Table 4:** Clustered logistic regression comparing AI vs human consultants A standard logistic regression with clustered standard errors was fitted to compare AI and consultant accuracy. The Wald z-statistic, Odds ratio (OR) and 95% confidence intervals (CI) are presented. Significance threshold: p < 0.05 (corresponding to |z| ≥ 2.58)

Comparison	Odds Ratio	95% CI (Lower–Upper)	Wald z	p-value
All five consultants vs AI	7.26	2.27 – 23.18	2.97	0.000817

## Discussion

This study compared the performance of an openly available AI model, ChatGPT 5, with that of experienced hand surgeons in identifying distal radius plate manufacturers from plain radiographs.

This task proved difficult for both humans and machines. The surgeons in this study, even with nearly 35 years of combined consultant experience, were only able to identify around half of the plates at best, reflecting differences in training or experience, overlap in design features across manufacturers and the inherent limitations of radiographs in distinguishing fine details or identifying features.

Nevertheless, clinicians consistently demonstrated superior recognition performance compared with AI. Across 52 radiographs, representing 10 major brands, all four consultants - even the ‘worst’ outperformed AI, which achieved an accuracy of just 5.8%. Although there was variability in human performance (13.5-46.2% correctly identified), when all consultants were considered together, a standard logistic regression with clustered standard errors showed that humans had over 7-fold higher odds of correct identification compared with the AI.

Although AI models can be trained for many aspects of medical image interpretation including hand and wrist fracture detection [17,18], the performance of an untrained model on this task is poor, underlying the limitations of general-purpose AI models when applied to specialised medical tasks [19]. Targeted training on datasets of orthopaedic implants therefore would be necessary to achieve clinically useful performance.

Limitations

Radiographs were only obtained in their AP projection and from open-access sources, so they may not fully reflect the range of quality or variation encountered in clinical practice. We only selected 10 manufacturers, and so inevitably many other smaller brands were excluded. 52 examples were selected (at least five of each). Our original power calculations did not anticipate that either group (but AI in particular) would identify so few plates correctly, and so this is probably an underestimation of the number required. Surgeons themselves often rely on a relatively small repertoire of implants determined by institutional preferences, training, and experience. Recruiting busy consultant hand surgeons is challenging; a larger number would not only have permitted us to calculate confidence intervals, but it would have potentially expanded the familiarity with a greater number of implants. Finally, the AI model evaluated was not specifically designed for implant recognition, which likely contributed to its low accuracy. This is a rapidly moving field, and our study only represents a snapshot in time; were it to be repeated in just 6 or 12 months, the results would likely be different.

## Conclusions

Volar locking distal radius plates are commonly used in the treatment of fractures or following corrective osteotomies. Recognising the plate manufacturer from plain radiographs is important when planning hardware revision, removal or periprosthetic fractures. In this study, even experienced hand surgeons struggled to identify 10 commonly used plating systems (13-46% correct). Nevertheless, they remained considerably superior to the performance of ChatGPT 5 on the same task (< 6%). Well-trained, custom-built AI systems are required to help in orthopaedic hardware recognition and, in time, are expected to become a useful tool in this domain.
